# Intern preparedness for the CanMEDS roles and the Dunning-Kruger effect: a survey

**DOI:** 10.1186/s12909-019-1836-z

**Published:** 2019-11-14

**Authors:** Detlef Richard Prozesky, Mmoloki Cornelius Molwantwa, Oathokwa Nkomazana, Masego Baitseng Kebaetse

**Affiliations:** Faculty of Medicine, University of Botswana, Private Bag UB, 0022 Gaborone, Botswana

**Keywords:** Undergraduate medical education, Medical internship, Programme evaluation, CanMEDS framework, Dunning-Kruger effect

## Abstract

**Background:**

The purpose of this study was to determine whether the first cohort of graduates from a new undergraduate medical programme in Botswana were adequately prepared for internship.

**Methods:**

The authors surveyed 27 interns and 13 intern supervisors on site, who rated intern preparedness for 44 tasks using a previously validated instrument. Tasks were grouped according to the seven roles of the physician in the CanMEDS framework and Cronbach α values confirmed internal consistency. To determine the direction of differences between intern and supervisor ratings for tasks Likert scale ratings were treated as interval data and mean scores calculated. Rating frequencies for each role were compared using the χ^2^ statistic. Reasons for differences between intern and supervisor ratings were explored by determining correlations between scores using the Spearman ρ statistic, and analysing qualitative data generated by the questionnaire.

**Results:**

Preparedness for all seven roles and the majority of tasks was found to be between ‘Fairly well prepared’ and ‘Well prepared’. The ratings for four roles (Medical expert, Communicator, Collaborator, Professional) differed statistically, but not for the three others (Leader, Health advocate, Scholar). Interns rated their proficiency higher than their supervisors for the tasks in six roles; for the ‘Professional’ role intern ratings were mostly lower. Correlations between intern and supervisors scores were only significant for three roles (Medical expert, Communicator, Collaborator). Qualitative data provided further insights into the reasons for these associations.

**Conclusions:**

Intern preparedness for tasks and roles varied but was generally satisfactory. Based on the analysis of the data seeming discrepancies in between interns and supervisor ratings were investigated and explanations are offered. For three roles the data indicate that their component tasks are understood in the same way by interns and supervisors, but not for the other roles. The Dunning-Kruger effect offers a plausible explanation for higher intern scores for tasks in six of the roles. For the ‘Professional’ role differences between interns’ internal, individual understanding and supervisors’ external, group understanding may explain lower intern scores. The fact that respondents may understand the tasks they rate differently has implications for all research of this nature.

## Background

Intern preparedness for medical practice has been the subject of discussion and research in medical education over the past decades. The term ‘intern’ refers to a recent medical graduate undergoing a period of supervised professional practice. Determining the preparedness of graduates of a new medical programme to practise as interns is a valued outcome metric for medical programmes [1]. The World Federation for Medical Education recommends that a medical school must ‘analyse performance of cohorts of students and graduates in relation to its mission and intended educational outcomes, curriculum and provision of resources’ [2]. In this sense ‘analysis’ is required so that successes are identified – and especially shortcomings, which can then be subsequently addressed.

In terms of scope, studies have dealt with a single institutions and programmes as well as multiple institutions - ranging from two institutions [3], several institutions [4–6] to an entire country [7–10]. There has also been comparison of two cohorts of the same programme such as where a traditional programme is being replaced by a new one with a problem-based ethos [4, 11–13], or one in a rural setting [14], or those comparing interns who had or had not undergone preparatory short courses [5]. In cases where the effect of curricular innovations was studied the results could be positive [13, 15]; in one study interns from a rural programme were judged to be better prepared for district hospital internship [14]. Other studies comparing innovative and traditional programmes revealed little or no difference in the resulting preparedness for clinical performance, but positive differences were found in areas of focus for the new programmes such as ethics and law, interpersonal skills, self-directed learning, health system functioning and collaboration, and negative ones in (for example) understanding of disease processes [11–13]. Beyond formal programmes, there has also been evaluation of the effectiveness of preparatory courses or ‘boot camps’ in preparing graduates for internship, whether general ones [16–19] or those with a particular focus such as surgery or paediatrics [5, 20, 21].

Studies have investigated both general and specific aspects of preparedness. For instance some studies have evaluated how well interns are prepared against official standards such as General Medical Council recommendations in the United Kingdom [10, 22, 23]. Where preparedness was measured against national standards, interns were sometimes not achieving them [23]. On the other hand, other studies have focused on the effect of programmes to prepare students for specific competencies such as disaster preparedness [24], emotion regulation [25], memorable ‘firsts’ [26], infant lumbar punctures [27], basic medical procedures [28], career preparation and guidance [3], vaccination [9], health advocacy (residents/registrars in this case) [29], a particular internship rotation [30], or developing a professional identity [6]. Additionally, studies have assessed the effect of entire undergraduate programmes on interns’ feelings of competence [7, 8, 31, 32]. When evaluating how well an entire undergraduate programme had prepared new interns overall, reports varied from reasonably well prepared [4, 13] to inadequately prepared [8, 31, 32]. In such cases, deficiencies such as those relating, handover, planning treatment, pain management, communication were identified [33] – also managing stress [25]. Such instances pointed to remedial action needed in undergraduate training.

Overall, studies have tended to be conducted in developed countries although there is emerging literature from African contexts [18, 32, 34]. In many cases the study population consisted of the interns themselves [3, 17, 18, 27, 28, 30], and in other studies their supervisors as well [10, 12, 13, 19, 22, 32, 35]. In most studies interns were approached at some stage in the course of their internship years. In some cases the study focused on final year students, investigating their prospective feelings of preparedness [16, 36]. Other studies investigated preparedness just before or immediately upon entry into internship [9, 19, 24, 27, 33], a few months after entry [12, 13, 15], before and after completing an internship rotation [30] or only afterwards [35], and after completing the entire internship [6, 31].

Studies have used qualitative approaches including interviews [14, 15, 25, 26, 30, 32, 37, 38], focus group discussions [26, 32] and diaries or audio-diaries [12, 26, 37]. Researchers have made us of self-administered questionnaires with space for additional qualitative comments, often in a survey design [20, 25–27, 30, 37]. Questionnaires were administered electronically (e-mail, online e.g. using SurveyMonkey) [3, 5, 9, 10, 29, 34, 36], by post [4, 7, 8, 12, 31], and in person [16–18, 20, 21, 24, 28, 35]. In a few cases, specific mention was made of validation processes where a previously validated instrument was used [10, 34], or the validation process for a tailor-made one was described [4, 13, 29]. Skills were sometimes observed [20, 21, 27]. Available records were also used, such as official forms [13, 19] and logbooks [26]. In some cases evaluations tracked cohorts of interns in longitudinal studies [11, 16, 33, 36].

This review of the literature revealed several challenges relating to evaluation of intern preparedness. Firstly, while it is common for studies to use intern self-evaluation with respect to their performance, it seems the validity of self-ratings was seldom questioned or taken into account in arriving at conclusions. Secondly, emerging evidence suggest that reliability can be enhanced through multi-source feedback, that is, through repeated observations and by a numbers of evaluators as an alternative to using single observations [39]. Thirdly, studies have tended to treat ordinal data obtained from Likert type questions as interval data for the purposes of analysis. Finally, in studies where both interns and supervisors gave ratings, supervisors’ ratings were higher than interns’ in some cases [11, 19, 35] and lower in others [22, 32]; this varied because in several cases interns and their supervisors observed different aspects of preparedness [10, 12, 13]. For instance, a systematic review of physician self-assessment compared with observed measures of competence showed that out of the 20 included studies 13 showed little, no, or an inverse relationship, and only seven demonstrated positive associations [40]. The tendency of some interns to over-rate, and others to under-rate themselves, can be possibly explained by the well-established Dunning-Kruger effect [41, 42].

In this article we describe research to determine the preparedness of the first cohort of graduates of a problem-based undergraduate medical programme in a middle-income country. We evaluated intern performance according to the interns’ own perceptions together with those of their clinician supervisors, using a parallel instrument. To conceptualise intern competency we investigated specific competencies as well as how they indicate competency for the seven CanMEDS roles [43] which our Faculty of Medicine has recently used as part of a framework for evaluating the MBBS programme. We also explored the possible role of the Dunning-Kruger effect on self-evaluation data.

## Methods

### Context

The first group of medical graduates of the new Bachelor of Medicine, Bachelor of Surgery (MBBS) programme at the University of Botswana completed their internship at the end of 2015. The programme is outcomes-based and uses problem-based learning as its main learning strategy throughout its five year duration. Towards the end of the internship period we realised that this was an opportunity not to be missed – we needed urgently to carry out an evaluation of their performance as interns, and to attempt to link this performance to the sum of their experiences in their undergraduate programme. The study objective was therefore to determine the extent to which the undergraduate MBBS programme at the University of Botswana prepared graduates to function effectively as interns.

### Study design and data collection

A survey study design was used, with information gathered from interns and their supervisors. The study population consisted of 35 interns and 20 physicians who had supervised them during internship. An anonymous questionnaire for interns with quantitative and qualitative elements was used, with permission from the University of Stellenbosch [34] which in turn had adapted it from an Australian study [4] to be more relevant to the local situation. The instrument was again modified slightly (two items added and three excluded) to suit our programme in Botswana. In addition a parallel questionnaire was prepared for supervisors (the source instrument was for interns only) since we believed it would add to the validity of the study: interns assess their individual experience (an ‘internal’ perspective), whereas their supervisors report on the interns they have worked with as a group (an ‘external’ perspective). The final instrument required both groups of respondents to grade preparedness for 44 routine internship tasks on a five level Likert type scale (Table 1) and to provide qualitative comments on tasks for which preparation was felt to have been good or insufficient. It was piloted with five interns and their supervisors, at a site where interns who had trained at other universities, and were therefore not eligible for inclusion in the research, were based. A few minor corrections to the instruments were made. It was administered in English since English is the language of secondary and tertiary education in Botswana and graduating students are fluent in the language, as are the doctors supervising the interns.

**Table 1:** Rating scale for internship preparedness: interns and their supervisors

1 = not prepared	Intern	I did not know how to do this/I did not feel prepared to do this, even with supervision
	Supervisor	The interns appear not to know how to do this/do not seem prepared to do this, even with supervision
2 = a little prepared	Intern	I was rather unsure of how to do this/I needed someone to guide me through the process
	Supervisor	The interns seem rather unsure of how to do this/need someone to guide them through the process
3 = fairly well prepared	Intern	I was fairly sure of my ability/I was willing to try with some help
	Supervisor	The interns seem fairly sure of their ability/are willing to try with some help
4 = well prepared	Intern	I felt that I knew how to do this/I could do this, but would have liked to have someone check my work
	Supervisor	The interns seem to know how to do this/can do this, but still want someone to check their work
5 = fully prepared	Intern	I knew how to do this really well/I felt able to do this well without any assistance
	Supervisor	The interns know how to do this really well/are able to do this well without any assistance

Ethical approval for the research was obtained from the Institutional Review Board of the University of Botswana. As explained above we collected the data near the end of the internship year (with the evident danger of pollution of understanding by learning undergone during the year) but there was also a logic to this delay, since interns would only have completed all the internship rotations by that time and some of the questionnaire items were specific to particular internship disciplines. The interns and their supervisors were visited in their workplace in three geographical internship hubs in the country and requested to participate voluntarily in the survey. The response rate was 77% for interns and 65% for intern supervisors. Each respondent was given a code and the data from each questionnaire entered into Excel spreadsheets. The entered data were checked and cleaned. Statistical calculations were performed using Excel and the Social Science Statistics website (https://www.socscistatistics.com/).

### Data analysis

The 44 tasks in the questionnaire were grouped according to the seven roles given in the well-known 2015 version of the CanMEDS framework [43]. We introduced this second level of analysis because it would help us to evaluate the interns’ preparedness not only for individual tasks but also for roles that are locally and internationally considered to be important. One of the authors who is familiar with CanMEDS was tasked with studying the CanMEDS document and allocating each task to the role which seemed to fit best. This was not always straightforward; for example the task ‘Evaluate the impact of family factors on illness’ could potentially be allocated to any of three CanMEDS roles, so the role in which the wording in the CanMEDS competencies most closely corresponded to that of the task was selected. After two iterations of this process the group of researchers approved the allocation. The result was as follows (Table 2):

**Table 2:** The CanMEDS roles and associated tasks in the study questionnaire

Role in the CanMEDS model	Tasks
1. Medical expert	10 tasks
2. Communicator	8 tasks
3. Collaborator	6 tasks
4. Leader	4 tasks
5. Health advocate	5 tasks
6. Scholar	4 tasks
7. Professional	7 tasks

The quantitative data from interns and supervisors were analysed as follows:

1. Frequency distributions for the ratings given by interns and supervisors for each task were determined, and summarised for each role. The percentages of ratings for each task and each role were calculated.
2. For both sets of respondents Cronbach α values were determined for the group of tasks in each role, to determine the internal consistency of the tasks in a role. One task ‘Function effectively in a resource constrained environment’ was excluded from the ‘Leader’ role since its inclusion resulted in an unacceptably low α value for the ‘interns’ group. The subsequent results are given in Table 3. There was now acceptable internal consistency in the way tasks were grouped in the roles.
3. To compare summarised ratings given by interns and supervisors for a role the two sets of five rating levels were compared using the χ^2^ test – the ratings being ordinal not interval. This was done to establish the degree to which interns and their supervisors differ in their ratings of roles.
4. The direction of differences in rating frequencies can be problematical since sets of ratings (even given as percentages) do not always show the direction of differences clearly, nor do they make it possible to estimate the size of a difference. To gain an understanding of the direction and size of these differences the scores were treated as interval data (using the allocated values from ‘1’ to ‘5’). This practice is also observed in other studies using Likert type scales [5, 12, 29]. In the text the results of this operation are referred to as ‘mean scores’.
5. Having completed Step 1 of the analysis we noted varying patterns of differences between intern and supervisor ratings for tasks and roles. In an attempt to understand these differences better the correlations between the mean scores for tasks within each role were also determined, using the Spearman rank correlation test. This was used since the Shapiro-Wilk test showed that none of the 14 datasets was normally distributed, with negative skewness in most cases.

**Table 3:** Cronbach α values for tasks in the CanMEDS roles for the intern and supervisor groups

Role	Interns	Supervisors
1. Medical expert	0.90	0.87
2. Communicator	0.87	0.90
3. Collaborator	0.84	0.89
4. Leader	0.71	0.88
5. Health advocate	0.87	0.89
6. Scholar	0.74	0.90
7. Professional	0.82	0.85

We considered threats to validity inherent in the chosen instrument. The ideal would have been to observe interns in their daily practice, using for example workplace-based assessment tools such as MiniCEX and DOPS, and to follow this by discussions about the degree to which undergraduate training had contributed to the observed performance. This method would however have been very time-consuming and the Hawthorne effect could be expected to operate [44]. The validity of the survey tool had received attention in the two previous studies in which it had been used, but reducing the observation of complex performance to a set of numbers is still a very serious threat to validity. We attempted to enhance the validity by piloting to ensure comprehension and by extending its use to supervisors as well as interns (thereby triangulating perceptions).

A relatively small amount of qualitative data were collected from comments invited about intern tasks for which preparation had been good or not. These were analysed using Miles and Huberman’s suggested process of display, reduction and conclusion drawing [45].

## Results

### Quantitative findings

The results of intern and supervisor ratings are given as mean scores, rather than frequency distributions of ratings which would have been too cumbersome. These mean scores are given in Table 4.

**Table 4:** Mean scores for each of the 44 tasks

	Interns		Supervisors	
	Mean	SD	Mean	SD
1. ‘MEDICAL EXPERT’ ROLE				
Carry out basic ward procedures	4.70*	0.72	4.08*	0.86
Carry out a comprehensive physical examination	4.26*	0.86	3.92	0.76
Draw up a comprehensive assessment of patients	4.07*	0.73	3.69	0.95
Appropriate use of diagnostic procedures in adults	3.89	0.70	3.56	0.88
Apply basic science knowledge to clinical conditions	3.78	1.01	3.46	0.88
Appropriate use of diagnostic procedures in children	3.78	0.85	3.38	0.92
Justify drug use based on mechanisms of action	3.67	0.96	3.50	0.80
Carry out basic surgical procedures	3.44	1.09	2.86†	0.69
Manage a woman in labour	3.22	1.01	2.25†	0.50
Handle most clinical emergencies	3.19	0.83	3.31	0.63
2. ‘COMMUNICATOR’ ROLE				
Treat each patient as an individual	4.12*	0.86	3.69	0.75
Provide education to patients	4.04*	0.90	3.50	0.67
Record clinical data systematically	3.93	1.07	4.00*	0.71
Counsel a distressed patient	3.67	0.73	3.31	0.63
Tell patients they have a terminal illness	3.52	0.98	2.91†	0.70
Manage ‘difficult’ (uncooperative) patients	3.22	0.70	2.92†	0.64
Deal with dying patients	3.15	1.20	3.25	0.45
Deal with relatives in distressing situations	3.15	0.82	3.00	0.85
3. ‘COLLABORATOR’ ROLE				
Approach senior staff for help if uncertain	4.41*	0.84	4.00*	0.71
Recognise own clinical limitations	3.93	0.87	3.69	0.85
Coordinate patient management with allied professionals	3.91	0.75	3.62	0.87
Appreciate group dynamics in a team environment	3.85	0.95	3.46	0.88
Take responsibility for patients’ clinical care	3.78	0.93	3.62	0.96
Sensitive to the needs of nursing staff	3.63	1.04	3.46	0.52
4. ‘LEADER’ ROLE				
Prioritise the day’s activities	3.59	0.84	3.31	0.85
Serve in administration/leadership roles if needed	3.48	1.09	2.82†	0.98
Actions to enhance healthcare facility effectiveness	3.38	0.94	3.62	0.51
Select drugs based cost, risks, benefits	2.85†	0.91	2.90†	0.74
5. ‘HEALTH ADVOCATE’ ROLE				
Appreciate the importance of patient culture/ ethnicity	3.81	0.68	3.75	0.75
Understand the interaction of social factors with disease	3.74	0.76	3.62	0.77
Evaluate the impact of family factors on illness	3.63	0.79	3.33	0.65
Discuss preventive strategies with patients	3.63	0.79	3.00	0.85
Able to respond to local community health care needs	3.33	0.78	3.00	1.07
6. ‘SCHOLAR’ ROLE				
Identify own learning needs	4.26*	0.86	3.62	0.77
Continually self-evaluate own performance	4.00*	0.73	3.08	1.00
Critically evaluate research related to practice	3.44	0.80	3.00	0.82
Invest time in developing own skills	3.41	1.08	3.67	0.65
7. ‘PROFESSIONAL’ ROLE				
Maintain attitudes appropriate to professional practice	4.15	0.95	3.62	1.04
Behave in a calm manner in difficult situations	3.56	1.01	3.38	0.65
Deal with own emotions when a patient dies	3.22	1.28	3.64	0.92
Able to approach ethical dilemmas	2.92†	0.80	3.00	0.63
Cope with stress caused by work	2.78†	1.01	3.08	0.90
Able to deal with medico-legal documentation	2.59†	1.19	2.91†	1.04
Balance own work and personal life	2.52†	1.19	3.36	1.12
ALL ROLES AND TASKS				
Average for all tasks	3.60	1.02	3.41	0.85

On the average interns judged themselves ‘Well prepared’ and above for nine tasks, and their supervisors for only two (all marked * in the table). Interns again judged themselves below ‘Fairly well prepared’ for five tasks (four of them in the ‘Professional’ role) whereas their supervisors made the same judgement for seven tasks (marked †). For all the other tasks the mean scores given by both interns and their supervisors ranged between ‘Fairly well prepared’ and ‘Well prepared’.

A summary of the ratings given by interns and their supervisors for each of the seven CanMEDS roles, and overall, is given in Table 5. The values are given in percentages for easier comparison. The significance of the differences was determined by conducting χ^2^ comparisons of the N values of each rating category for each role.

**Table 5:** Summarised intern and supervisor ratings for roles in %s, with χ^2^ comparison

Rating (%)	Not prepared		A little prepared		Fairly well prepared		Well prepared		Fully prepared		Comparison by χ^2^	
Source	Int.	Sup.	Int.	Sup.	Int.	Sup.	Int.	Sup.	Int.	Sup.	χ^2^	p
Role												
1. Medical expert	1.1	0	9.3	14.3	25.2	30.5	37.4	43.8	27.0	11.4	12.67	0.013
2. Communicator	0.9	1.0	13.5	10.1	30.2	47.5	35.8	37.4	19.5	4.0	17.43	0.0016
3. Collaborator	1.2	0	6.2	7.7	21.0	33.3	43.2	46.2	28.4	12.8	9.932	0.042
4. Leader	3.7	2.3	16.8	16.3	30.8	39.5	40.2	39.5	8.4	2.3	2.59	0.63
5. Health advocate	0	2.2	6.7	15.6	34.8	28.9	47.4	48.9	11.1	4.4	7.96	0.093
6. Scholar	1.9	2.1	6.5	12.8	26.9	36.2	41.7	44.7	23.1	4.3	9.29	0.054
7. Professional	11.2	2.4	18.6	17.1	31.9	36.6	25.0	36.6	13.3	7.3	10.02	0.040
All 7 roles	2.9	1.2	11.2	13.0	28.4	36.5	37.7	41.9	19.8	7.4	47.68	< 0.001

The ratings given by interns and supervisors for individual tasks and roles differed considerably. Overall and for four of the roles the differences in intern and supervisor ratings were significant at a level of *p* < 0.05, using the χ^2^ test for these ordinal data (Table 5). As explained in the Methods section the direction of differences between intern and supervisor ratings are difficult to interpret, so they were analysed further by mean scores. In an attempt to explain possible reasons for the differences, the correlations between mean scores for all the tasks in each role were also calculated (Table 6).

**Table 6:** Mean scores per CanMEDS role for interns and supervisors: comparisons and correlations

Role	Mean scores					Correlation	
	Interns (I)		Supervisors (S)		Difference: I-S	Spearman ρ	P
	Mean	SD	Mean	SD			
1. Medical expert	3.80	0.98	3.52	0.88	0.28	0.924	0.00013
2. Communicator	3.60	0.98	3.33	0.76	0.27	0.707	0.050
3. Collaborator	3.91	0.92	3.64	0.81	0.27	0.853	0.031
4. Leader	3.33	0.98	3.19	0.82	0.10	0	1
5. Health advocate	3.63	0.77	3.37	0.84	0.25	0.021	0.74
6. Scholar	3.78	0.94	3.36	0.85	0.42	−0.200	0.80
7. Professional	3.11	1.19	3.29	0.92	−0.18	0.643	0.12
All 7 roles	3.60	1.02	3.41	0.85	0.20	0.684	0.0000

**Fig. 1:**
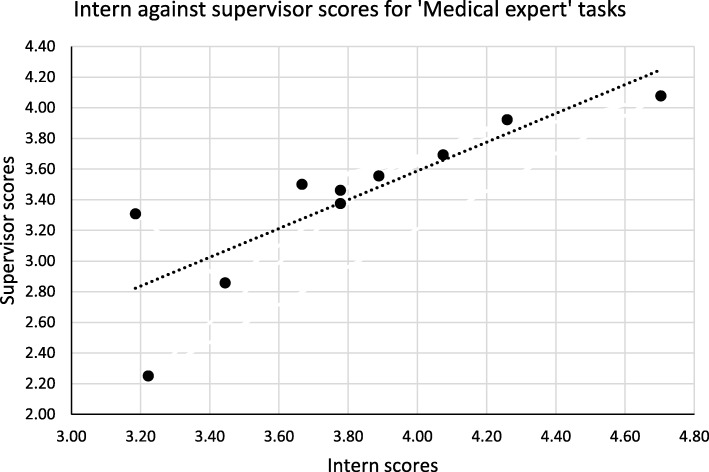
Comparing intern and supervisor mean scores for the ‘Medical expert’ role

**Fig. 2:**
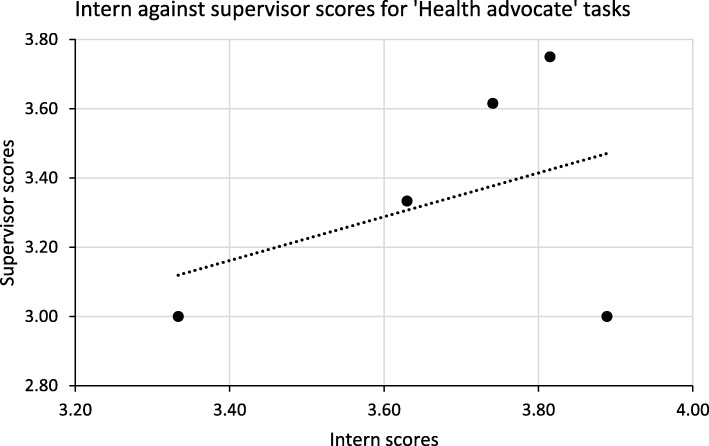
Comparing intern and supervisor mean scores for the ‘Health advocate’ role

Overall and for six of the roles the intern mean scores were higher than those of their supervisors; however for the ‘Professional’ role the supervisor mean score was higher. Varying patterns of the mean scores of interns and supervisors emerge: from large differences and significant correlations (the ‘Medical expert’ role), to large differences and poor correlation (the ‘Scholar’ role), to small differences and poor correlation (the ‘Leader’ role). Two of these examples are illustrated in Figs. 1 and 2 below.

Figure 1 demonstrates good correlation between intern and supervisor scores, whereas the opposite is shown in Fig. 2.

### Qualitative findings^1^

Analysis of the qualitative data revealed two overarching themes. The first was the number of propositions made concerning each role, as an indication of the importance of that role in the minds of respondents – noting that the data arose from general questions about preparedness or lack of it, and not from questions directing respondents to the seven CanMEDS roles. The spread of propositions is shown in Table 7.

**Table 7:** The number of qualitative propositions about the seven roles

Role	Number of propositions made by:	
	Interns N (%)	Supervisors N (%)
1. Medical expert	76 (52.1)	52 (65.0)
2. Communicator	11 (7.5)	3 (3.8)
3. Collaborator	17 (11.6)	1 (1.3)
4. Leader	16 (11.0)	3 (3.8)
5. Health advocate	1 (0.7)	5 (6.3)
6. Scholar	9 (6.2)	6 (7.5)
7. Professional	16 (11.0)	10 (12.5)
TOTAL	146 (100)	80 (100)

Not surprisingly the majority of propositions related to the ‘Medical expert’ role: 52 and 65% of intern and supervisor propositions respectively. This role therefore seems to predominate in respondents’ understanding of the work of an intern – reflecting the reality of the CanMEDS diagram, which places the ‘Medical expert’ role in a central position with the other six roles in support of it [43]. The variation in attention given to the other roles by interns and supervisors respectively is also noteworthy: supervisors have little to say about the ‘Communicator’, ‘Collaborator’ and ‘Leader’ roles and interns equally neglect the ‘Health advocate’ role.

The second theme overall concerns similarities and differences between intern and supervisor propositions for the different roles. Overall about half of the propositions from both interns and supervisors were positive (relating to tasks for which preparation had been good) and half negative (relating to tasks for which preparation could have been better). However within each role this distribution was much more varied.

### The ‘Medical expert’ role

Supervisors generally agreed that interns’ ‘general approach to patients was excellent’. Interns reported having ‘good history and physical exam skills’ which was confirmed by supervisors: ‘They are able to do comprehensive history taking and physical examinations’ although there was room for improvement in ‘detailed history taking especially the history of present illness’. Interns and supervisors concurred that interns do well in ‘choosing the necessary investigations’ and being ‘able to interpret investigations and apply them to patient care’. Interns reported ‘being able to assess systematically patient, highlighting the important factors necessary for diagnosis’, but whereas one supervisor reported that ‘they are able to reach to relevant diagnoses’ another felt that interns lacked ‘familiarity with and awareness of cognitive errors in clinical reasoning’. Supervisors stated that ‘interns take responsibility for the clinical care of their patients’ and interns reported being able to ‘manage patient throughout that entire ward stay’. There were however a number of caveats. Supervisors and interns commented on interns’ lack of ability in ‘management of critical patients’ – ‘they could have been better prepared in the handle of most clinical emergencies.’ Interns themselves made particular mention that they lacked skill in ‘basic obstetric procedures and management of common complications’; in ‘end of life care/palliation’ and ‘dealing with death of a patient’, and ‘justifying drug uses on basis of mechanism of action’. Supervisors felt that interns could have been better prepared in ‘management of non-communicable diseases (diabetes, hypertension emergencies, epilepsy)’ and ‘guideline-guided clinical practice’. Interns and supervisors generally concurred that interns were skilled in ‘doing simple clinical skills like IV cannula, NG tube, urinary catheter, taking bloods’ – also chest drains, lumbar punctures and ‘basic diagnostic procedures in both children & adults.’ On the other hand some supervisors mentioned that ‘there is a need to improve on how to carry out basic surgical skills.’ Concerning theory underpinning clinical practice the messages were mixed. Interns reported being good at ‘applying basic sciences and translating concepts into medical practise’ whereas a supervisor noted that ‘their use of the pre clinical knowledge especially for anatomy and physiology + pathology was a bit low.’

### The ‘Communicator’ and ‘Collaborator’ roles

The ‘Communicator’ role did not seem to be a major concern for supervisors, with few and relatively superficial comments being made: ‘conduct clinical discussions well’ and ‘very good clinical notes’. For interns on the other hand this was a more important issue with many commenting on the major theme, counselling patients. Several interns reported feeling inadequate about ‘counselling of patients + their families’ and ‘breaking bad news to pts as well as relatives.’ For the ‘Collaborator’ role the principal theme was that of teamwork. Remarkably only one supervisor commented at all, stating that interns are ‘very professional with good teamwork’. All the other propositions came from interns. They had mostly positive opinions about their ability as team players: ‘being part of a team & playing my role as an intern’ and ‘working as a team and supporting each other’. A smaller, negative intern theme was that of support: ‘More encouragement from the system. We are reprimanded for all the bad things but there is no positive reinforcement, doing ward rounds by yourself in instances where there is NO M. O or specialist from your team present’.

### The ‘Leader’ and ‘Health advocate’ roles

Propositions about the ‘Leader’ role dealt with the theme of taking responsibility. In the hospital setting some interns report that they ‘take responsibility of the team’s needs during daily ward rounds’ but others did not feel prepared well enough for ‘leading a team on your own, doing ward rounds by yourself’. One intern reported that s/he ‘also impressed my supervisors when it comes to organisation’. In a wider, district-level setting interns are uncertain about ‘moving to the rural-urban areas in Botswana and being expected to function as well as I did in Gaborone/referral hospitals’, and comments from a supervisor reflect this: ‘they are unable to site any roll-models of competent and fullfilled general practitioners and thus do not see that type of career as an option for them.’ The ‘Health advocate’ role received only one comment from interns, an appreciation of her/his ‘knowledge of HIV as its a major public health issue in our setting’. The few supervisor comments were about defects: ‘the importance of public health interventions methods/skills need to be emphasised’ and interns being unable ‘to look at the system’s problems as challenges which they can help their country overcome rather than obstacles which are poised to overcome them.’

### The ‘Scholar’ and ‘Professional’ roles

Concerning the ‘Scholar’ role the main theme that emerged was that of an evidence-based approach to practice. Here there was a considerable variation of opinions between and within the intern and supervisor groups. Supervisors stated that interns were ‘very knowledgeable, well read’ and ‘able to do critical appraisal of scientific papers and apply the knowledge on their day to day clinical practice’ – but also that ‘they have knowledge but needs to be more confident and forthcoming with information’. Some interns felt skilled in ‘applying evidence based clinical research in management and approach to patients’ but others lacked confidence in ‘critically appraising medical research papers’. The ‘Professional’ role has two key components: professional behaviour related to practice, and a commitment to the practitioner’s own health. In both aspects preparedness was variably (and mainly negatively) judged by the two sets of respondents. In terms of professional behaviour one supervisor reported in summary that intern performance is ‘widely variable with some interns performing very well from the outset and even roll modeling positive behaviour while others giving the impression that they had rarely, before internship, been in an environment where … they were challenged to perform at the highest standards’. Supervisors were also of the opinion that ‘there is need for extra emphasis on medico-legal and ethics of medicine’ and interns similarly report uncertainty in ‘handling medico-legal documents’. In terms of practitioner health several interns report feeling inadequate in ‘managing work stress and making time for my social life’ and achieving ‘emotional balance in the work place when there is too much to do and when patients are difficult.’ Although not listed as a task interns also reported that they lack skill in ‘financial management for personal benefit’ and a supervisor reported that interns have ‘uncertainty about the future … lack of a clearly defined career path.’

## Discussion

The principal objective of the research was to determine whether graduating interns and their supervisors believed that the new undergraduate medical programme at the University of Botswana had adequately prepared them to work as interns in the hospitals where they had been placed. The mean overall scores that both interns and their supervisors gave to the selected tasks were 3.61 and 3.40 respectively – in other words between ‘fairly well prepared’ and ‘well prepared’, and well short of the ‘fully prepared’ level. This was also true of each individual role (Table 5).

As shown in the introduction studies of intern preparedness internationally are characterised by their variety of objectives, design and instruments, spatial orientation, study populations and findings. The present study shares characteristics of other studies of this nature but is identical to none of them. The current study investigated a new programme and not an established one [10]. It investigated the outcome of one programme, rather than comparing two programmes [4, 11]. The survey was done on completion of the internship year [7], rather than early on in it [12] or even before embarking on it [33]. Data were gathered from interns and their supervisors [10, 19, 35], and not only from the interns themselves [18, 28, 30]. The study investigated overall competence [31, 32], rather than competence in one specific area [3, 21]. It focused on interns’ ability to carry out relevant tasks grouped according to the CanMEDS roles; other studies investigated competence in relation to national guidelines [22, 23]. In this study interns rated themselves more highly than their supervisors did [5], rather than the other way around [11, 19]. In common with the findings of other studies it reveals broad overall competence in tasks and roles, but with specific shortcomings [34]: in this case surgical procedures, managing labour, communicating with patients with terminal illness, managing uncooperative patients, serving in leadership roles, and selecting drugs in a cost-effective way. A further special characteristic of the present study is that it was conducted in a middle-income Southern country.

The interpretation of the findings of the study raises important questions, in relation to the congruence of intern and supervisor ratings in studies of this kind. The supervisors rated interns’ ability to carry out tasks lower than the interns themselves did for Roles 1 to 6. For Roles 1, 2 and 3 there is good internal consistency, a significant difference in rating and significant correlation between mean scores. In such cases it appears likely that interns and supervisors had the same conception of tasks (and the roles overall), but the supervisors were more strict in their evaluation (see Fig. 1). The qualitative data relating to the ‘Medical Expert’ role support this: both interns and supervisors made positive comments about interns’ basic skills but supervisors noted more areas where improvement was needed. The research done by Kruger and Dunning [41] indicates that persons who are less skilled tend to rate their performance in tests more highly than they should – their actual performance belies their judgment of it. The most likely explanation for this phenomenon is that such persons not only lack knowledge and skill, but they also lack the metacognition required to judge their performance as inadequate – the very knowledge and skill that they lack they also need to judge their performance. Relatively inexperienced interns would tend to lack the metacognitive ability they need to evaluate their own performance. The systematic review of Davis et al. about the accuracy of physician self-assessment compared with observed measures of competence shows that out of 20 comparisons 13 showed little, no, or an inverse relationship and only seven demonstrated positive associations [38]. The worst accuracy in self-assessment was found with the least skilled and the most confident – a seeming parallel with the Dunning-Kruger effect [41].

The situation in Roles 4, 5 and 6 on the other hand is different. There is good internal consistency between ratings for both interns and their supervisors, but the combined ratings of interns and supervisors are not significantly different neither is the correlation between their mean scores. The wording in the intern and supervisor questionnaires was practically identical and both were pre-tested, so the most likely explanation seems to be that within each group members have similar conceptions of the tasks (cf. the good Cronbach α values) which however differ from the conceptions of the other group – hence the poor correlation (see Fig. 2). The qualitative data bear this out: there are few areas concerning these three roles in which interns and supervisors concur. Even in the first three roles discussed above the data show that, in spite of a large degree of common understanding, supervisors and especially interns note some specific and different areas of intern weakness. This seems to be an almost inevitable weakness of studies of this nature, with few observers with different backgrounds making observations. In their study of the reliability of multi-source feedback Moonen-van Loon et al. have shown that to achieve high reliability several occasions for observation by a relatively large number of assessors is needed, and that non-physicians’ scores for the ‘Scholar’ and ‘Health advocate’ CanMEDS roles and physicians’ scores for the ‘Health advocate’ role had a negative effect on composite reliability [39] – the latter a seeming parallel with the findings of this study. But even for these three roles the Dunning-Kruger effect may be operating overall.

Williams, Dunning and Kruger [42] have also demonstrated that there is a curvilinear relationship between objective performance and self-evaluations of ability. The difference between actual and self-perceived ability is greatest in highly unskilled persons, and this difference gets less the more skilled or knowledgeable the person is. Medical graduates are unlikely to be ‘highly unskilled’ or ‘highly lacking in understanding’ so the difference between their estimation of performance and their actual performance (as judged by their more skilled and experienced supervisors) should not be too great. This is in fact what our study has found: for Roles 1 to 6 the difference in mean scores is a mere 7.6% overall.

The ‘Professional’ role seems to be a special case. In five of the seven tasks interns rated themselves lower than their supervisors did. We propose two possible explanations. Interns necessarily have an ‘internal’, individual, personal view of their competence, whereas supervisors have an ‘external’ view of the competence of a group of interns. For tasks like ‘Deal with own emotions when a patient dies’, ‘Cope with stress caused by work’ and ‘Balance own work and personal life’ interns know that they should appear to cope. They may be rated highly by their externally observing supervisors, while being aware of their own struggles and feelings of inadequacy and therefore rating themselves lower. The qualitative data support this conclusion: supervisor comments dealt with the ‘professional behaviour’ component of this role, externally observed, whereas interns commented primarily on negative aspects of their personal experiences of the ‘practitioner health’ component. Another possible explanation is Kruger and Dunning’s finding that very highly skilled persons tend to be less confident about their relative performance than they should be [41].

## Study limitations

This study has several limitations. Although the response rates were fair the number of interns and supervisors is relatively small; so too the amount of qualitative data produced by the questionnaires. The data collection instruments that were used were originally designed for different setting, and the data collected with them were not intended to be analysed according to the CanMEDS model – future research would do well to use a more specifically designed instrument. Collecting data at the end of the internship period carried with it the danger of contamination by learning undergone during the year; however the questionnaire contained a clause emphasising the need for respondents to reflect specifically on the effect of undergraduate training on performance as interns. As Moonen-van Loon et al. point out [39] judgements made from single observations, as in this study, should be interpreted cautiously. Finally the study provides information for a very specific situation, so may have limited application to other programmes elsewhere.

## Conclusions

The study objective was achieved – we now know that our new MBBS programme prepared interns reasonably well. We also know of specific deficits in their performance which need to be corrected. The study has helped us to gain more insight into a process of data analysis which was initially carried out rather mechanistically, copying what was used in similar studies. During data analysis we were brought to question the pattern of differences between intern and supervisor ratings which sometimes seemed contradictory. We attempted to explain these by referring to the Dunning-Kruger effect, and by considering the different ways in which interns and their supervisors may have experienced and conceptualised tasks: interns evaluating themselves personally and internally, and supervisors evaluating a collective of interns externally. Remedial activity in the MBBS programme is currently underway; to evaluate its effect we need to repeat this study with a new group of interns after a suitable delay. Additional qualitative data in such a study should help to determine whether our explanations about the nature of differences in the data hold water, and may therefore be important in other studies – the fact that respondents may understand the tasks they rate differently has implications for all research of this nature.

## Data Availability

The datasets used and/or analysed during the current study are available from the corresponding author on reasonable request.

## Abbreviation

MBBS: Bachelor of Medicine, Bachelor of Surgery

## Footnotes

1 Grammatical and spelling errors in quotations used in this section have not been edited.
